# Climate of Nebraska—Cerebral and Bowel Affections, Etc.

**Published:** 1871-03

**Authors:** Noble Holton

**Affiliations:** Falls City, Neb.


### CLIMATE OF NEBRASKA—CEREBRAL AND BOWEL AFFECTIONS, ETC.

By NOBLE HOLTON, M.D., of Falls City, Neb.

Prof. N. S. DAVIS—*Dear Sir:*—My lot was cast in the above-named place the last of July, 1870, the principal object being to test the climate in its effect upon the health of my wife. She has been suffering from an hereditary rheumatism of a chronic and sub-acute character, for 18 years, principally affecting the large joints. This town is situated in latitude 40 north, and on rolling and well-drained prairie, and 30 to 50 feet higher than the adjacent Nemeha river bottom, just south of it. This bottom is from a mile to two miles wide, and generally partly covered with water, and a heavy growth of vegetation. It is not so muddy as to hinder teams crossing it at any time, or in any place, except it is on account of extreme high water, or when the frost is going out in the spring.

The stream is a good-sized mill-stream, and having a quick current with many good chances for water-power. The soil is a sandy loam, very productive, with a porous under-soil, rapidly absorbing moisture, and capable of producing prolific crops, even in seasons of extreme wet or drouth, under proper cultivation.

The town has a population of 1,000, and, like other western towns, is a lively place, and quite rapidly increasing in population with a preponderance of youngerly people and children.

All these preliminaries have been written so that readers might judge what influence they had in producing what I am about to relate.

When I arrived here, the country was suffering from quite an extreme drouth, and also from a high temperature, it being no unusual thing for the mercury to reach 90° or 100° F. On Sunday, July 31, 1870, I was called, in consultation with Dr.

Shaw, to see a child, about a year old, of Esq. F.'s. It had been troubled for a week with serous diarrhœa, light-colored and fetid discharges, not so profuse as to have caused much emaciation, with occasional vomiting. The reason for alarm, when I was called, was, that on the 30th the vomiting had suddenly become so frequent, the child could not retain anything on its stomach—either drinks, food, or medicine—and was very restless. Upon careful observation, the vomiting was seen to be an ejection of the contents of the stomach at a gulp, and this would occur more promptly when the child's body was held perpendicularly. This led me to examine the brain more carefully, when the pupils were found to be unequally dilated, the large veins of the neck turgid, and the anterior fontanelle fuller than natural, together with increased heat of head.

From these symptoms, the conclusion was that there existed hyperæmia, or, perhaps, inflammation of the brain, and that this peculiar vomiting was caused by the brain disease, and not by irritation or inflammation of the stomach. Whether this was true or not, we agreed upon the following treatment:

℞ Potass. bromid.,------------------------℈j.

Aqua distil.,---------------------------℥j.

M.

And give a dose sufficient to make one grain of the bromide every four hours; also,

℞ Bismuth s. n.,------------------------gr. j.

Soda bi-carb.,-----------------------gr. ss.

Hydrarg chlor. M.,------------------gr. ¼.

M.

And give a powder every four hours, alternately. Cold sponging to the head was also recommended. Warmth to the extremities and beef-tea for nourishment, well salted, with urgent instructions to keep the child as quiet as possible and recumbent. Under this treatment it promptly improved, and when the active symptoms were relieved, the tannate of quinine and other tonics completed the cure. The week I arrived here, there were six deaths of similar cases, and the people were thoroughly alarmed.

Following the case related, there were two attacks in children of moderate diarrhœa, with occasional vomiting, which readily improved under ordinary treatment; but on Friday I saw a child with Dr. Hanna who was having fetid, fecal, billious and mucous discharges, and a little vomiting, and had been sick four days. This child was taking calomel in small doses, combined with bismuth, opii., ipecac., and rheubarb, with tannate of quinine in the remission.

It appeared to be doing so well there was no change made, but in a few days brain symptoms came on, and the child died. Not having an opportunity of seeing it again, I cannot tell what the treatment was.

On the 19th of August I saw a child, two and a-half years old, with Drs. Hanna and Shaw, suffering from summer complaint three weeks, with vomiting, loss of appetite, and, consequently, emaciation.

This case was accompanied with well-marked remissions and exacerbations, and was put upon quinine as a general remedy, under which he seemed to improve for a few days, but he again grew worse, when he was given iron, cinchonia, and bismuth, alternating with bromid. potass.

He, however, gradually failed, brain symptoms set in, and he died Sept. 12th.

On the 22d of August I saw, with Dr. Shaw, a child, one year of age, of Mr. N.'s, who had been troubled with attacks of vomiting and diarrhœa, occasionally, for two months, but now the vomiting was very frequent, and diarrhœa mucus, with tenesmus on passing the discharges. He was ordered turpentine emulsion, alternated with potass. bromid.

℞ Pul. gum arabic,---------------------------

White sugar,—āā,----------------------ʒij.

Spts. turpen.,----------------------------

Tinc. opii.,—āā,------------------------ʒij.

Mint water,----------------------------℥ij.

M. Dose, 15 drops every four hours.

℞ Potass. bromid.,-----------------------℈j.

Aqua distil.,-----------------------------℥j.

M. And give sufficient to make a grain of the bromide every four hours.

Dr. Shaw, by mistake in writing the directions, ordered a teaspoonful of the emulsion, and, if the tenesmus was severe, a teaspoonful and a-half.

Consequently, in the morning early, I was called to see the child immediately, for it was thought to be dying. The child was found to be under the exaggerated effects of opium, and a drop of Ext. Bellad. fl'd was given at once, sinapisms to extremities, the bromide to be continued, with cold to the head, and emulsion discontinued.

The active symptoms gradually subsided, and a course of quinine, followed by ferrated elixir calisaya completed the cure.

During July, August, and September, there were fifteen deaths of children under two and a-half years of age, all terminating with cerebro-spinal disease, in town and surrounding neighborhood.

About the first of September, the hot, dry weather gave place to a cold, rainy period, which continued for six weeks. On the 8th of August I was called to see an infant child of Mr. C.'s, who was taken with mild diarrhœa and occasional vomiting, but readily recovered, so that no further attention was given it until the 8th of September, when the diarrhœa was more obstinate, but not considered alarming till the 12th, when it exhibited unmistakable brain symptoms. It was put upon the potass. bromid., and the proper adjuncts, and in 24 hours seemed very much better; but next day the symptoms rapidly grew worse, and it died in two days.

My observation in this case convinced me that there was not only loss of tone of the whole system, and loss of the saline and plastic properties of the tissues, from the long continued and intense heat, but also that there was a powerful septic poison operating to destroy life by attacking the nerve centres. These cases were called cholera infantum, but in a practice of twenty-five years I had not seen it terminate with so trifling exhausting discharges, and so invariably with brain symptoms. The sulphites had been used to some extent, on general principles, but now they were used to neutralize the supposed septic influence. The sulphite of lime was selected in those cases where the digestive organs were irritated, as the soda might increase the trouble.

When, on the first of September, the weather became cool and wet, the attacks of summer complaint were superseded by attacks of typho-malarial fever, the use of the sulphites could be prosecuted no further in the cases of summer complaint. These cases were preceded by a few days of illness, when a well marked chill, followed by fever and a remission, ushered in the fever.

These were the symptoms of remittent fever, but when carefully observed, the remission was not complete, and there was a peculiar dingy appearance of the face, with dulness of intellect, indicating a more profound disturbance of function than would be seen in a remittant.

The chills and remission would grow less and less in succeeding days, a serous diarrhœa would come on, with dry tongue, red at tip and edges, brown in the middle, sordes on the teeth and lips, a tympanitic abdomen, and mild delirium at night.

In most of these cases there was a rose rash on the chest or abdomen, appearing at different time from the attack, in different cases from four to twelve days.

The treatment was a mixed one: mercurials at first to stimulate the secretions, sulphites as antiseptics, quinine as an antiperiodic, to meet the malarial element, and the turpentine emulsion for the more particularly typhoid condition. Under this treatment I know of only one death, and that one was caused by congestion of the brain, ushered in by a severe chill, and followed by collapse, like a pernicious intermittent. This occurred in the fourth week of the disease. The fever usually continued two weeks, but varied from nine days to four weeks, but patients gaining strength very slowly after that. South and southeast of town is the Nemeha river bottom, from a mile to two miles wide, and during the hot, dry weather the wind was often from that direction, and the town, on a gentle elevation, extends to the bottom. This is the only source of malaria apparent in the neighborhood, and, according to authorities, would be expected to produce autumnal or malarial fevers, but in what way did it operate to produce such mortality among children, invariably terminating in cerebro-spinal disease? And, can a change of temperature and moisture cause a typho-malarial fever in adults? I know of no way of accounting for it except by the addition of a septic poison of an unusual character to assist it, for it is true the undersoil is porous and the atmosphere dryer than east of the Missouri river.

During these months there were cases of disease, exhibiting all grades of disturbance of the digestive organs, dysenteric, choleric, and neuralgic. A case of a man 65 years old, who was attacked, while threshing on Saturday, Aug. 20th, with cholera morbus, and called Dr. Wilson, but as he continued to grow worse, I was called on Tuesday evening.

At the time, he was vomiting, and purging watery discharges, and extremities cold and cramping, pulse small and frequent. He was ordered bismuth and morphine, to be taken immediately after a spell of vomiting, about once in two hours, with sinapisms to feet, over the stomach, and along the spine; also, artificial warmth. Saw him again next day, but there was no reaction, and he gradually failed and died next night. He had been ordered beef-tea, well salted, for nourishment, and milk porridge. The relatives informed me Dr. W. gave him a dose of pills, after which he grew worse.

Mrs. S. called me Sept. 19, on account of purging and vomiting, accompanied with great depression. Under appropriate treatment the vomiting and serous diarrhœa were arrested, but the irritation extended to the colon, causing dysenteric evacuations. By modifying the treatment, to meet this indication, followed by quinine and other tonics, she fully recovered, much to the surprise of her friends and neighbors.

In the latter part of the year there was an apparent lack of tone, a debilitated condition, exhibited even in those who were not actually sick, and mothers who were lying-in had lingering labors generally from inertia of the womb. Now, were the extreme hot and dry months of the summer the cause of this septic poison and all these symptoms, is the question?

I believe they were. In reference to the effect of the climate upon the health of my wife, I am happy to state she has been gradually improving.

The tissues appear to be condensed, the joints assuming a more natural appearance, and the soreness subsiding, and the use and elasticity returning. The dry, bracing, and, perhaps, slightly alkaline, air from the plains may be credited for this effect. I also consider the climate well adapted to asthmatic and catarrhal diseases on the same principle. I have known a number of persons subject to these complaints entirely relieved by a residence here.

